# Identification of Clinical Measures to Use in a Virtual Concussion Assessment: Protocol for a Mixed Methods Study

**DOI:** 10.2196/40446

**Published:** 2022-12-22

**Authors:** Keely Barnes, Heidi Sveistrup, Mark Bayley, Mary Egan, Michel Rathbone, Monica Taljaard, Shawn Marshall

**Affiliations:** 1 School of Rehabilitation Sciences Faculty of Health Sciences University of Ottawa Ottawa, ON Canada; 2 Bruyere Research Institute Ottawa, ON Canada; 3 Clinical Epidemiology Program, Ottawa Hospital Research Institute Ottawa, ON Canada; 4 Kite Research Institute Toronto Rehabilitation Institute University Health Network Toronto, ON Canada; 5 Division of Physical Medicine and Rehabilitation Temerty Faculty of Medicine University of Toronto Toronto, ON Canada; 6 Division of Neurology, Department of Medicine Faculty of Health Sciences McMaster University Hamilton, ON Canada; 7 School of Epidemiology and Public Health University of Ottawa Ottawa, ON Canada; 8 Department of Medicine University of Ottawa Ottawa, ON Canada

**Keywords:** telehealth, virtual care, concussion, mild traumatic brain injury, assessment, examination

## Abstract

**Background:**

Workplace concussions can have a significant impact on workers. The impact of concussion symptoms, combined with challenges associated with clinical environments that are loud, bright, and busy, create barriers to conducting effective in-person assessments. Although the opportunity for remote care in rural communities has long been recognized, the COVID-19 pandemic has catalyzed the transition to virtual assessments and care into the mainstream. With this rapid shift, many clinicians have been completing remote assessments. However, the approaches and measures used in these assessments have not yet been standardized. Furthermore, the psychometric properties of the assessments when completed remotely using videoconference have not yet been documented.

**Objective:**

Through this mixed methods study, we aim to (1) identify the concussion assessment measures clinicians are currently using in person and are most relevant to the following 5 physical domains: neurological examination (ie, cranial nerve, coordination, motor, and sensory skills), cervical spine, vestibular, oculomotor, and effort assessment; (2) document the psychometric properties of the measures identified; (3) identify measures that appear feasible in a virtual context; and (4) identify practical and technical barriers or challenges, facilitators, and benefits to conducting or engaging in virtual concussion assessments.

**Methods:**

This study will follow a sequential mixed methods design using a survey and Delphi approach, working groups with expert clinicians, and focus groups with experienced clinicians and people living with concussions. Our target sample sizes are 50 clinicians for the Delphi surveys, 4 clinician-participants for the working group, and 5-7 participants for each focus group (roughly 6-10 total groups being planned with at least two groups consisting of people living with concussions). The results from this study will inform the decision regarding the measures that should be included in a virtual assessment tool kit to be tested in a future planned prospective evaluation study.

**Results:**

The study is expected to be completed by January 2023.

**Conclusions:**

This mixed methods study will document the clinical measures that are currently used in person and will identify those that are most relevant to assessing the physical domains impacted by concussions. Potential feasibility of using these measures in a virtual context will be explored.

**International Registered Report Identifier (IRRID):**

DERR1-10.2196/40446

## Introduction

### Background

Concussions or mild traumatic brain injuries affect thousands of Canadians every year [1]. Workplace injuries account for a notable portion of nonsport concussions [2]. Terry et al [3] reported that approximately 25% of adult concussions occur at work. Workplace injuries resulting in concussions pose a significant challenge for employers and insurers while disrupting the lives of the workers due to greater recovery times compared to non–work-related concussions [4]. In general, roughly 10%-20% of individuals who sustain a concussion experience symptoms lasting beyond the one- to two-week typical recovery time frame [5,6]. In the workplace context specifically, prolonged symptoms lead to reduction in productivity at work or even disability, which in turn has an economic impact on people, companies, and government agencies [3]. Although it is apparent that there is a need for further support and research in the workplace concussion rehabilitation field, a focus on the evaluation of concussion will be an important starting point to individualizing care.

Assessing symptoms and function after concussion presents clinicians with challenges due to the complex and diverse symptom presentation and a lack of sensitive and reliable clinical assessment measures [7]. Symptoms may be physical (such as dizziness, balance issues, headaches, neck pain, and vision difficulties), emotional (such as irritability and disinhibition) or cognitive (such as memory and concentration difficulties) [7-11]. All the domains of concussion symptoms must be considered [8]. Currently, however, a gold standard test to evaluate all concussion symptoms does not exist. Commonly, a battery of tests and symptom self-reports are relied upon [7,12,13]. For example, the Balance Error Scoring System and the Sensory Organization Test are used to assess balance deficits following a concussion injury [14]. The King-Devick test and the Vestibular Ocular Motor Screen may be used to evaluate oculomotor deficits [14,15]. Furthermore, the psychometric properties of the tools involved are often underdeveloped, and clinical utility varies. There is an additional challenge with all assessments of injured workers because of access to compensation that may interfere with the validity of effort provided during the assessment [3]. Measures that allow the examiner to ensure that a valid effort was made to complete the assessment should be considered.

With the shift in clinical service delivery to virtual health care driven by the COVID-19 pandemic, the need for valid and reliable approaches to virtual concussion assessments has become more pressing. In addition, people living in remote areas or for whom travel is difficult due to comorbidities continue to have a great need for assessment that can be carried out at distance [16,17]. Such assessment would also be helpful for people whose symptoms are aggravated by travel and environmental factors such as noise and light [18].

Clinicians are currently completing virtual concussion assessments using a variety of clinical assessment measures; however, as with in-person assessments, there is no standardized approach to assessing adult concussions virtually. Resources have been developed to support completion of the virtual concussion examination, including a training manual for the examination and a living guideline that outlines considerations for the pediatric examination [19,20]. Comparison of the measures identified in these resources when administered in an in-person and virtual context has not yet occurred.

The psychometric properties of many of the assessment measures being used have not been established for use in person or virtually. Accelerated adoption of information and communication technology has been occurring globally to enhance service delivery during the COVID-19 pandemic; however, it is unclear whether the outcomes of assessments completed virtually are consistent with assessments performed in person [21,22]. It is, therefore, important to understand if the measures used to assess concussions in person could provide equivalent results when used virtually [23].

Overall, there are multiple clinical assessment measures being used by clinicians to assess the physical domains impacted by a concussion injury. There is no standardized tool kit of measures available with established psychometric properties that are relevant and feasible in a virtual context, nor are there guidelines outlining specific measures to use in the adult population [12-14].

### Objective

The objective of this mixed methods study is to produce a tool kit of measures that can be used in virtual assessments of concussion’s physical symptoms.

## Methods

**Figure 1:**
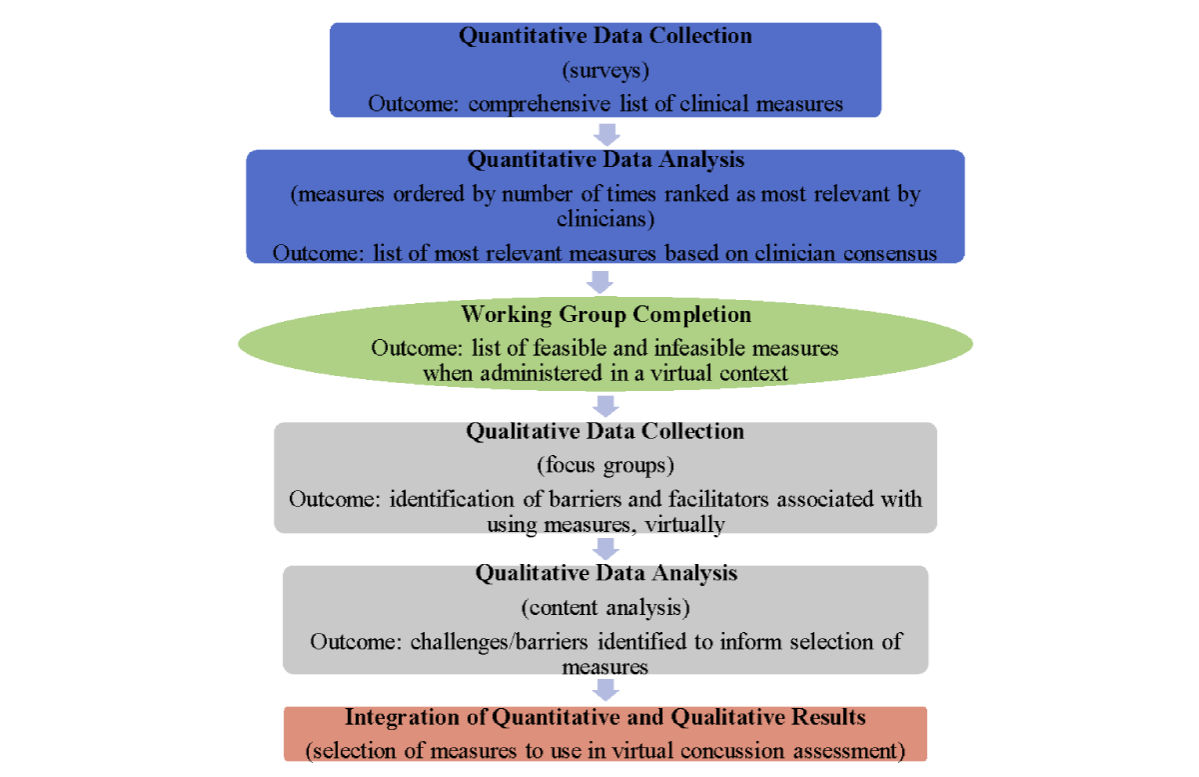
Sequential explanatory approach commencing with survey administration guided by the Delphi methods and working group completion (quantitative) followed by focus group completion (qualitative) to expand on and explain survey and working group results.

This study will follow a sequential mixed methods design [24] that will include a Delphi survey, a working group consultation, and focus groups. The methodological approach is outlined in Figure 1.

### Participants

#### Delphi Survey

Expert clinicians (ie, physiatrists, neurologists, sports medicine physicians, and physiotherapists) from across Canada will be identified through both regional and national professional and brain injury or concussion organizations and networks. Members of these networks represent an accessible and meaningful sample that will include many practicing clinicians with concussion assessment experience. The survey will be sent through these networks and associations by including the survey link in a newsletter or by sending the survey link through email to all members of the networks and associations. To ensure ‘experts’ are completing the survey, respondents will be asked to rate their concussion expertise using a 5-point Likert scale (“strongly not competent” to “strongly competent”). Any responses from participants self-reporting their competency as below 3 or “neutral” will be excluded. When needed, targeted emails will be sent to expert clinicians with publicly available contact information to ensure representation (minimum of 2 responses) of each profession.

#### Sample Size for Delphi Survey

Our target sample size is 50 clinicians, which we consider adequate to reach saturation on the types of clinical measures used in practice. This sample size is feasible based on an anticipated 25% response rate and assuming that approximately 200 clinicians will view the survey [25,26]. Clinicians who complete the first-round survey will be sent the second-round survey. It is expected that the second round of surveys will elicit a response rate of approximately 60% (~30 clinicians) [27].

#### Working Group Membership

A working group consisting of expert clinicians, including at least one neurologist, physiatrist, sports medicine physician, and physiotherapist, will meet to discuss the feasibility of virtual use of each measure identified in the survey. Members of the research team will be offered the opportunity to participate. Targeted emails to Canadian practicing clinicians will also be used to ensure representation from each clinical field. A final list of potentially feasible outcome measures will be identified in the working group, which will then be further explored in the focus groups.

#### Focus Group Membership

Focus groups consisting of 5 to 7 participants [28,29] will be conducted following the methodological framework outlined by Breen [30]. It is hypothesized that 6 to 10 focus groups will be adequate to reach saturation (2-3 groups consisting of people living with concussions who have experience with virtual assessment and 4-8 focus groups consisting of clinician-experts) [28,29]. Patient-participants who have attended a virtual concussion assessment will be identified and recruited from the Ottawa Hospital Rehabilitation Centre. The remaining focus groups will contain mixes of neurologists, physiatrists, sports medicine physicians, and physiotherapists. Participants who complete the Delphi surveys will have the option to express interest in participating in a focus group. Additional recruitment strategies include face-to-face recruitment at the Ottawa Hospital Rehabilitation Centre and through Ontario Workers Network clinics. Targeted emails to Canadian practicing clinicians will also be used.

### Procedures

#### Quantitative Delphi Survey Data Collection

**Figure 2:**
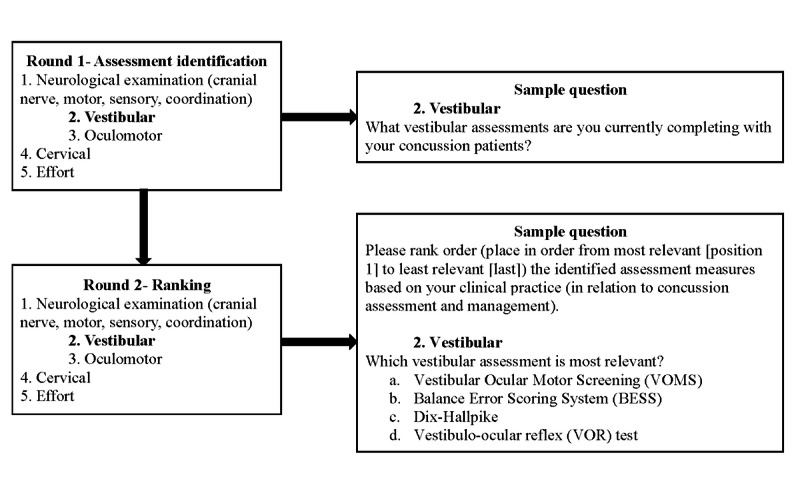
Clinical domains of interest for identification of assessment measures and sample questions for the vestibular domain for round one and two surveys. Similar questions were provided for each domain.

The survey process will follow a Delphi method, which aims to seek consensus among the participants [31]. In round one, clinicians will receive a link for participation, which includes the implied consent form and a demographic questionnaire with open-ended questions; the questionnaire will have free-text boxes asking participants to identify multiple outcome measures they currently use to assess each 5 domains of interest (Figure 2). The invitation to participate in the surveys will be undersigned by a physiatrist (SM), including the affiliation, to secure buy-in and encourage responses. Measures identified by at least 15% of all participants (eg, 8/50 participants) or at least 60% of participants from one profession will be retained for the survey in round two.

In round two, all participants from round one will be asked to rank order, according to perceived usefulness for their practice, the concussion assessment measures from each domain (ie, neurological exam, cervical spine, oculomotor, vestibular, and effort) retained from the first round (Figure 2). Prior to completing the second-round survey, participants will be invited to review reading materials with information on the retained measures, including information on sensitivity, specificity, and feasibility from the literature, when available.

#### Delphi Survey Questionnaire Administration

To increase response rates, reminder emails will be sent at 7-day and 14-day time points following the initial emails in the first and second rounds [25,30]. A universal level of consensus does not yet exist for the Delphi survey approach; however, various works have suggested a range of 51%-80% agreement [27]. For the purposes of this study, an above 51% agreement level for rankings of assessments has been set for the second round. Assessments that reach 15% in round one will be considered for clinical feasibility by the expert clinician working group.

#### Working Group to Derive Consensus on the List of Feasible Measures

A working group consisting of expert clinicians will meet over a videoconferencing platform, which involves bidirectional visual and audio communication technology, to discuss the current feasibility of the identified measures in a virtual context. Any measures deemed not feasible in the working group will be eliminated from further discussion in the focus groups. The final list of measures will be further explored in the clinician focus groups.

#### Qualitative Focus Group Data Collection

Focus groups with both clinician and patient-participants will be conducted. An interview guide with broad open-ended questions will be used as a prompt for the conduct of the focus groups. All focus groups will occur over a videoconferencing platform and will be audio and video recorded for later transcription. Multimedia Appendix 1 includes the semistructured interview guide for both patient-participants and clinician-participants.

##### Clinician-Participant Focus Groups

Prior to conducting the focus group, clinician-participants will be provided with available psychometric properties documented in the literature of the in-person measures that were identified from the second round of the surveys as well as descriptions of the assessments and instructions on how to complete the assessments. Clinician-participants in the focus groups will be prompted about the practical and technical issues associated with using each of the final measures. Barriers and facilitators associated with the assessments, including adverse events experienced or observed, will also be explored.

##### Patient-Participant Focus Groups

Patient-participants will be prompted to discuss the benefits and challenges associated with virtual concussion assessments based on their experiences.

### Data Analysis

#### Quantitative Analysis of Delphi Surveys

Clinical measures identified in round one of the Delphi surveys will be categorized into the preestablished domains (ie, neurological examination, cervical spine, vestibular, oculomotor, and effort assessment). Frequency counts of the measures will be used to identify most commonly identified measures [32], and consistency of wording for the description of measures will be documented. For example, some clinicians may describe the measure rather than providing the name of the measure.

The quantitative data obtained in the second round of surveys (ie, rank order of measures) will be analyzed descriptively (ie, summary statistics to demonstrate patterns in the data). Measures of frequency and agreement percentages will be calculated.

#### Qualitative Analysis of Focus Groups

NVivo will be used to organize the qualitative data analysis. Recordings from the focus groups will be transcribed verbatim. Content analysis will be used to analyze the focus group data [33]. Two research assistants will independently identify codes related to barriers or challenges, benefits, and facilitators associated with using each of the identified measures. Codes will be sorted into 2 levels of categories: categories related to assessment of specific symptoms or domains and categories related to virtual assessment in general.

### Ethics Approval

Ethics approval was obtained by the Ottawa Health Sciences Network Research Ethics Board (20210575-01H) in September 2021 followed by the Bruyère Research Institute Research Ethics Board (M16-22-006) and the University of Ottawa Board of Ethics (H-02-22-7611) in February 2022.

## Results

Survey administration and the working group have been completed. Focus group recruitment is underway. The final results of the surveys, working group, and focus groups will lead to the identification of clinical measures to use in a virtual assessment tool kit, which will be tested in a future planned evaluation study.

## Discussion

### Expected Outcomes

We presented a protocol for a mixed methods study to identify the most appropriate clinical measures to include in a virtual assessment. We hypothesize that the measures identified in the surveys will vary based on clinical profession. We anticipate reliability properties of the identified measures to range from moderate to strong (intraclass correlation coefficients=0.41 to above 0.81). It is anticipated that the working group and focus group discussions will lead to an understanding of some of the real and perceived barriers and facilitators related to participating in or completing a virtual concussion assessment. We expect patient-perceived challenges to relate to the technical issues with technology and clinician-perceived challenges to relate to the challenges associated with engaging in hands-on approaches in virtual care.

A toolbox of concussion physical assessments has been proposed by Matuszak et al [14], which includes measures to assess domains that are frequently impacted by a concussion injury; however, many of the physical assessment measures used in the concussion population have limited psychometric data. The proposed toolbox includes evaluation of vital signs, mental status, neurological examination (ie, cranial nerves, manual muscle testing, and reflexes), head and cervicothoracic evaluation, balance or coordination assessment, and vestibulo-ocular evaluation [14]. This toolbox is proposed for the in-person examination. However, many of the virtual resources and guidelines include similar evaluations [19,20].

Although telehealth has existed for a long time, the COVID-19 global pandemic has increased both its need and its use in delivering health care services [34]. The COVID-19 pandemic has accelerated the need for telemedicine-supported remote assessments and has pushed both clinicians and researchers to determine what aspects of medical care could be feasible in a telehealth context [34,35]. A scoping review by O’Neil et al [36] noted that videoconferencing could be a valid means to remotely assess patients with moderate to severe traumatic brain injury. In addition, according to Fjeldstad-Pardo et al [37], no adverse events have been identified during participation in telerehabilitation, indicating that it is a safe approach to deliver services. Specifically, telerehabilitation has been reported to be feasible and effective for the intervention and management of neurological patients [38]. There has been a significant increase in the use of virtual assessments, with a rapid transition due to the pandemic. This transition limited the ability to implement virtual care using a planned and organized approach. The transition was a reaction to the pandemic rather than a planned response [39]. Due to this reactionary response, clinicians have been forced to complete remote assessments with limited information on the accuracy and reliability of the measures used in these assessments.

It is, therefore, important to understand which assessments clinicians are completing both in person and virtually in the context of the concussion examination. A study by Tobler-Amman et al [40] found that correlation values were low when measures assessing similar constructs in people living with stroke were administered in person and virtually. Although clinicians thought the measures were assessing the same construct, the low correlation values indicate that administering the measures in the two different contexts may not provide the same information. Similarly, a study by Wang et al [41] on people living with Parkinson disease found that participants elicited a weaker reach when an assessment was completed virtually compared to in person. In the context of the concussion examination, understanding which assessments are being conducted and how the assessments are being conducted both in person and virtually is necessary to determine the equivalence of the assessments when administered in both contexts. Psychometric properties should be established in both in-person and virtual contexts of use to ensure accurate and reliable implementation in both contexts [23]. If the outcome measures used in person have poor documented psychometric properties or no documented psychometric properties, there may be additional challenges to validity and reliability when using the measure in a virtual context. It is important to gain an understanding of the properties of the clinical measures and their ability to produce equivalent results to the in-person assessment to inform decision-making in terms of their use in practice and their potential use in the virtual context [42].

With the increase in need for the uptake of telehealth in rural and remote areas, and now globally due to the COVID-19 pandemic, standardization of a feasible virtual concussion assessment is needed. The identification of the clinical measures that are most relevant and contribute the most reliable information in an adult concussion examination and the identification of the barriers and facilitators associated with using these specific measures in a telehealth context is an important first step to lead to this standardization. The results of this study and the future planned prospective evaluation study will be published and disseminated to targeted end users and networks.

### Strengths and Limitations of Methodological Approaches

An important strength of the Delphi method is that it is a useful approach when lack of clarity exists, which is likely the case in the concussion assessment field [43]. It is apparent that although the Delphi approach has many strengths, it also has some limitations. Generally, the Delphi approach lacks agreed-upon standards or consensus [43].

Focus groups have been reported to be beneficial when participants are in different geographical locations [30], which will occur in this project due to the need to gain input from various clinicians and from people living with concussions to inform the follow-up studies. Some important limitations of focus groups include the following: the group setting nature of the focus groups may discourage participants from sharing their input; similarly, one single participant may dominate the focus group discussion (ie, overpower other participants) [30].

### Conclusions

This mixed methods study will identify the assessment measures that are currently being used in person to evaluate people living with concussions. This study will further identify the barriers and facilitators as well as the challenges and benefits associated with using the identified measures, virtually. Psychometric properties documented in the literature of the in-person measures that will be discussed in the focus groups will be described. Results from this study will inform the selection of measures to include in a virtual assessment tool kit, which will be tested in follow-up studies. It is apparent that there are multiple gaps in the clinical assessment of concussion. There appears to be a need to further explore and expand on the understanding of the measures used to assess individuals experiencing prolonged symptoms in person; however, there is currently a need to explore the use of these measures in a virtual context. The limitations of the COVID-19 pandemic have highlighted the need for a rapid change in service delivery to virtual means, and although limited information exists regarding concussion assessment in a virtual context, remote assessments are currently being conducted in clinical practice, and therefore, need to be better understood.

## Appendix

Multimedia Appendix 1 Interview guide.

Multimedia Appendix 2 Peer reviewed by Workplace Safety & Insurance Board (WSIB) Grants Program (Toronto, Ontario, Canada).

## Data Availability

Data sharing is not applicable to this study, as no data sets were analyzed during the study.
