# Supplementary material for: Identification of Clinical Measures to Use in a Virtual Concussion Assessment: Protocol for a Mixed Methods Study
Source: JMIR Res Protoc. 2022 Dec 22;11(12):e40446. doi: 10.2196/40446 (PMC9816949; doi:10.2196/40446)
Supplement: Multimedia Appendix 1 [file resprot_v11i12e40446_app1.docx]

**Clinician-Participant Focus Group Interview Guide**

1. General conversation about what clinicians experience as barriers and facilitators to virtual assessment of concussion symptoms.
2. Have you experienced any adverse events while completing a virtual assessment? (If so, could you discuss it?)
3. For each of the identified measures from the second Delphi round and working group, the following questions will be asked:

a. Of the identified [vestibular] assessments, which of these do you think would work best in your practice?

a. What are some barriers to using the measure in a virtual concussion assessment?

i. What are some practical issues associated with using the measure in a virtual context?

ii. What are some technical issues associated with using the measure in a virtual context?

b. What are some facilitators to using the measure in a virtual context?

i. What are some practical facilitators associated with using the measure in a virtual context?

ii. What are some technical facilitators associated with using the measure in a virtual context?

**Patient-Participant Focus Group Interview Guide**

1. Please tell me about the virtual assessments you’ve participated in.
2. Based on your experience, what are some challenges/barriers you faced when completing a virtual assessment?

a. Practical barriers?

b. Technical facilitators?

1. What are some benefits/facilitators you experienced?

a. Practical facilitators?

b. Technical facilitators?

1. Do you have any recommendations that could improve virtual assessments?
2. Do you have any other feedback based on your experiences with virtual assessment?
