# Supplementary material for: Identification of Clinical Measures to Use in a Virtual Concussion Assessment: Protocol for a Mixed Methods Study
Source: JMIR Res Protoc. 2022 Dec 22;11(12):e40446. doi: 10.2196/40446 (PMC9816949; doi:10.2196/40446)
Supplement: Supplementary file 2 [file resprot_v11i12e40446_app2.pdf]

Workplace Safety & Insurance Board

Head Office:
200 Front Street West
Toronto, Ontario, Canada M5V 3J1
1-800-387-0750 | **TTY** 1-800-387-0050 | **F** 1-888-313-7373
wsib.on.ca

May 20, 2021

Dear Dr. Marshall,

Re: Proposal submitted to the 2021 WSIB Grants Program, project entitled,*"Validation of virtual neurological assessment in the treatment and rehabilitation of concussion patients"*

Thank you for your patience as we finalized the evaluation of your proposal. The WSIB is pleased to inform you that your proposal has been conditionally approved for funding subject to the terms and conditions set out in the attached Grant Agreement and is conditional on the execution of a signed Grant Agreement.

Please review the enclosed Agreement carefully, including all Schedules and Attachments. Note, Schedule D (the Grant Application) has not been attached to the Grant Agreement to minimize the file size. A PDF copy of the Grant Application will be attached to the Grant Agreement prior to finalizing the required document in order to obtain the signatures.

In addition to the Grant Agreement, please review, complete and sign the **Research Agreement** in order to access WSIB data. Please forward this information to grants@wsib.on.ca.

Please note that this letter is CONFIDENTIAL. You and your institution must withhold any private or public sharing of any information contained in this letter until 1) a Grant Agreement which is satisfactory to the WSIB in its discretion is executed and 2) the WSIB has publicly announced the 2021 grant recipients except for disclosures to employees of your institution with a need to know in order to review and execute the Grant Agreement.

Additional comments from the review panels have also been included for your reference.

Congratulations! I look forward to working with you and your team in the months ahead.

Matthew Mayer
Manager, Strategic Research

SECTION 1: WORK PLAN

- This study has high value impact as it presents a potentially valuable resource which could have significant benefits for workers who might otherwise have issues accessing care or for those in remote regions of the province
- Addresses an urgent need to assess the reliability and validity of telemedicine approaches to neurological assessment
- Directly applicable to the work of the WSIB and could dramatically revolutionize the way the WSIB does these assessments
- Results could have positive customer service value (less time and energy intensive for IP)
- The work plan is well articulated and aligns well with the project objectives and is supported in terms of feasibility and staffing
- Concern around the ambitiousness of the proposal both with respect to sample size and clinician time required

SECTION 2: SCIENTIFIC MERIT

- This study has direct relevance to value for money as it relates to ways in which WSIB offers services – OHAP
  - Negating the need to travel will cut down on travel costs and on the overall load and resulting fatigue for the IP
- Very complete literature review highlighting important gaps, especially applications of virtual testing outside of acute sports injuries
- Potential limitations of the approach are the difficulties of assessing level of effort and the need for sensor-based technology for fine assessment of neck range of motion and other physical tests
- Concern that only English speaking individuals will be utilized which does not represent the make-up of WSIB workers

SECTION 3: RESEARCH DESIGN/METHODOLOGY

- The study is well designed and supported by a strong disciplinary team
- Concerns about the feasibility to recruit 240 participants required and the ability to engage 10 clinicians in the assessment protocol given the current pandemic context, the additional strains placed on the health care system, and the reliance upon participants attending the clinic for in-person assessment
  - It is unclear if the in-person research testing sessions will be prioritized in light of the current demands placed on the healthcare system by the pandemic (e.g., assessment of post-COVID neurological symptoms; treatment of post-COVID patients)
  - The rate of remuneration for a physician specialist appears low ($150 per hour) and may limit the ability to secure sufficient clinicians for the 240 assessment sessions required
- The applicants mention age as a factor impacting on the effectiveness of virtual assessment, however, little treatment is given to this potential confound in the proposal
- Limitation of the proposal is that intersectional identity factors such as sex/ gender and SES are not considered nor is coverage given to equity, inclusion and diversity

SECTION 4: BUDGET

- Budget provides detailed tasks and expenses, staffing hours, meeting costs, data analysis, etc.
- The remuneration for physician specialist time appears to be low
  - If the assessments are performed primarily by allied health professionals, the generalizability of these findings might be limited
- The budget shows that participants will be attending a three-hour session at the clinic ($14 in additional funds is provided for parking), however, this is unclear in the proposal itself

SECTION 5: KNOWLEDGE TRANSFER & PRACTICAL APPLICATION OF RESULTS

- The KT plan is well articulated and comprehensive
- The knowledge transfer and application of results is very relevant to the WSIB worker population, particularly those located in remote regions or those with issues accessing care
- Multiple methods and platforms will be used to ensure knowledge transmission and update
- The findings and potential tools identified will be of significant utility to not only the WSIB but other stakeholders provincially and nationally
- Steps are provided to engage the WSIB at various project design and implementation stages
    - The researchers will require WSIB coordination to help recruit clinicians

SECTION 6: OTHER CONSIDERATIONS

- The applicants are very renowned in their field with the required knowledge, experience, and training to complete this work and are supported by a strong interdisciplinary team
- Note was made of the potential for ‘technological risk’, and although a plan is in place to mitigate, it remains possible that technological issues may impact the study negatively
